# Production and purification of higher molecular weight chondroitin by metabolically engineered *Escherichia coli* K4 strains

**DOI:** 10.1038/s41598-020-70027-9

**Published:** 2020-08-06

**Authors:** S. D’ambrosio, A. Alfano, E. Cassese, O. F. Restaino, S. Barbuto Ferraiuolo, R. Finamore, M. Cammarota, C. Schiraldi, D. Cimini

**Affiliations:** Department of Experimental Medicine, Section of Biotechnology and Molecular Biology, University of Campania L. Vanvitelli, via de Crecchio 7, 80138 Napoli, Italy

**Keywords:** Industrial microbiology, Applied microbiology, Biotechnology, Polysaccharides

## Abstract

The capsular polysaccharide obtained from *Escherichia coli* K4 is a glycosaminoglycan-like molecule, similar to chondroitin sulphate, that has established applications in the biomedical field. Recent efforts focused on the development of strategies to increase K4 polysaccharide fermentation titers up to technologically attractive levels, but an aspect that has not been investigated so far, is how changes in the molecular machinery that produces this biopolymer affect its molecular weight. In this work, we took advantage of recombinant *E. coli* K4 strains that overproduce capsular polysaccharide, to study whether the inferred pathway modifications also influenced the size of the produced polymer. Fed-batch fermentations were performed up to the 22 L scale, in potentially industrially applicable conditions, and a purification protocol that allows in particular the recovery of high molecular weight unsulphated chondroitin, was developed next. This approach allowed to determine the molecular weight of the purified polysaccharide, demonstrating that *kfoF* overexpression increased polymer size up to 133 kDa. Higher polysaccharide titers and size were also correlated to increased concentrations of UDP-GlcA and decreased concentrations of UDP-GalNAc during growth. These results are interesting also in view of novel potential applications of higher molecular weight chondroitin and chondroitin sulphate in the biomedical field.

## Introduction

Chondroitin sulphate (CS) is an anti-inflammatory drug, largely used for the treatment of osteoarthritis, and recent novel applications, spanning from the use as cosmeceutical for its hydrating and protective functions and in medical devices for eye pathologies and urinary tract infections, to the development of biomaterials in the field of regenerative medicine, are present^1–6^. Another emerging application field regards the design of CS-based delivery systems, due to its high biocompatibility, biodegradability, non-immunogenicity and low toxicity. In fact, since it can be degraded by the colon micro-flora, it was investigated as material for colon-specific drug-delivery systems^7^. Due to its ability of binding CD-44 receptors that are overexpressed on cancers cells, CS was also used to decorate drug-gene loaded nanocarriers for tumor targeting strategies^8,9^.

The capsular polysaccharide (CPS) produced by *Escherichia coli* K4, possesses a chondroitin-like structure composed of alternating, β-linked, glucuronic acid (GlcA), and *N*-acetyl-d-galactosamine (GalNAc) and of a fructose residue linked on the C3 of GlcA. As CS, also unsulphated biotechnological chondroitin produced from recombinant *E. coli* K4, showed anti-inflammatory activity in an osteoarthritis-like in vitro model^10^,moreover, it recently also demonstrated a potential in the treatment of neurodegenerative diseases protecting cells by different cellular stresses as amyloid aggregates, advanced glycan end products (AGEs) and reactive oxygen species (ROS)^11^.

The already established and also the new potential applications of biotechnological chondroitin and of CS might benefit from polymers with a higher molecular weight (Mw), that could show improved viscoelastic properties, further broadening the application fields. It is therefore interesting to investigate whether changes at the biosynthetic level in *E. coli* K4 can be used to manipulate the chain length of the produced capsular polysaccharide.

We previously characterized in shake flasks different recombinant strains that overexpress genes involved in the last steps of UDP-GalNAc and UDP-GlcA biosynthesis, namely *kfoA*, *kfoF*, *pgm*, and *galU*^12^. All strains demonstrated increased CPS titers, however major improvements were encountered in those overexpressing the UDP-glucose dehydrogenase, encoded by *kfoF*, either alone or in combination^12^. Previous studies in other hosts also demonstrated that UDP-glucose dehydrogenase is a rate limiting enzyme in the production of GAG and GAG-like polysaccharides^13–17^ and in recombinant *Bacillus subtilis* its overexpression increased the Mw of the produced polymer by 35%; however, in this case the polymer size was almost reduced by half when scaling the process from shake flasks to 3 L bioreactors, due, according to the authors, to the shear stress caused by mechanical agitation^13^. Also the downstream purification process can affect the size of the polysaccharide produced. We recently developed a small-scale downstream method, that utilizes ultrafiltration (UF) and anion exchange chromatography, to purify the capsular polysaccharides directly from the fermentation broth^18^. This allowed the determination by size exclusion chromatography-triple detector array (SEC-TDA) of the Mw of the fructosylated K4 CPS and of the heparosan, produced respectively by *E. coli* K4 and *E. coli* K5 strains^18^. However, this purification method still lacks, in the path towards relevant medical applications, a step to obtain a carbon backbone that is identical to that of CS, which is the elimination of the GlcA-linked fructose residues present in the K4 CPS.

The aim of the present work was, therefore, to investigate whether the previously described^12^ metabolic engineering strategies affected the molecular weight of the produced polymers, and to potentially correlate this to the modification of intracellular metabolite pools in order to elucidate the regulatory network of K4 CPS quantity and quality on multiple cellular levels. Fed-batch processes up to the 22 L scale were used to evaluate the performance of the different strains and to determine polymer size in potentially industrially applicable controlled fermentation conditions. We next investigated operating conditions that would allow fructose removal while minimizing uncontrolled, concurrent polymer degradation. Therefore, a preliminary protocol exploiting short-term hydrolysis combined with UF, ethanol precipitation, and chromatography was also developed to obtain defructosylated, high molecular weight biotechnological chondroitin, with a low endotoxin content and a final purity of about 93–96%.

This work represents a first step towards the production of higher molecular weight chondroitin and eventually chondroitin sulphate whose potential applications have not been investigated so far.

## Materials and methods

### Medium and strains

The medium used for bioreactor experiments consisted of a defined salts medium (KH_2_PO_4_ 2 g/L; K_2_HPO_4_ 9.7 g/L; Na_3_C_6_H_5_O_7_ 0.5 g/L; (NH_4_)_2_SO_4_ 1 g/L; MgCl_2_ 0.1 g/L) supplemented with 10 g/L of glucose as the main carbon source and 2 g/L of yeast extract, as additional nitrogen source (Oxoid, Ontario). The concentrated feed used for fed-batch experiments contained 350 g/L glucose and 70 g/L of yeast extract. The wild type strain used in all experiments was *E.coli* K4 serotype O5:K4:H4 (CCUG 11307), purchased from the Culture Collection University of Goteborg. The genetic characteristics and intended metabolic targets of recombinant strains^12^ used in the work are reported in Table 1. For all recombinant strains kanamycin was supplemented in the solid (50 µg/mL) and liquid media used for all experiments.

**Table 1:** Description of the strains used in the work.

Strain or plasmid	Description	Metabolic targets	Reference or source
*E. coli* O5:K4:H4	Wild type strain	Not applicable	CCUG 11307
pCR-XL-TOPO	*E. coli* cloning vector pUC origin; Kan^r^	Not applicable	Invitrogen
EcK4rv_1	K4 derivative; contains XL vector with *kfoA* under the control of p*gapAP1*	Increase of UDP-GalNAc biosynthesis	^12^
EcK4rv_2	K4 derivative; contains XL vector with *kfoF* under the control of p*gapAP1*	Increase of UDP-GlcA biosynthesis	^12^
EcK4rv_3	K4 derivative; contains XL vector with *kfoA and kfoF* under the control of p*gapAP1*	Increase of UDP-GalNAc and UDP-GlcA biosynthesis	^12^
EcK4rv_5	K4 derivative; contains XL vector with *pgm*, *galU* and *kfoF* under the control of p*gapAP1*	Increase of UDP-Glu and UDP-GlcA biosynthesis	^12^

“Metabolic targets” indicates the intended biological objective of the genetic modification. UDP-Glu (UDP-Glucose).

### Bioreactor experiments

Fermentation experiments were performed in 2.5 and 22 L Biostat CT plus and Biostat C fermenters, (Braun Biotech International, Sartorius Group, Germany). Seed cultures were prepared by inoculating a 20% v/v glycerol stock solution into 200 mL of medium in 1-L baffled shake flasks. After about six hours the flasks were used to inoculate the main culture. Fed-batch experiments lasted 23 h, and were conducted at 37 °C, at a constant pH of 7.5, maintained by addition of 30% (v/v) NH_4_OH or 30% (v/v) H_2_SO_4_ and at 30% air saturation. When all glucose initially present in the medium was exhausted, after about 6–7 h of growth, the concentrated solution was fed with a profile that ranged between 2 and 3 g/L of carbon source per hour (Supplementary Fig. S1); the same profile was used for all strains on both bioreactors. Pulse additions of kanamycin restoring a concentration of 50 µg/mL were performed every two h in the first 12 h of growth to keep a high selection pressure and reduce plasmid loss. Polysaccharide production at the end of the process (23 h) was established by High Performance Capillary Electrophoresis (HPCE), as previously described^18^. This value was used for the calculation of the volumetric productivity (r_p_). For the determination of the concentration of lipopolysaccharide (LPS) during the course of the purification process, if necessary samples were concentrated (up to a theoretical concentration of 20 g/L of chondroitin to avoid viscosity issues) and analysed by HPCE^19^. The concentration of glucose and of organic acids produced were evaluated by HPLC^12^. Biomass samples were collected during the mid-exponential phase to extract UDP-sugars, briefly, as previously described^20^, 0.12 g_cdw_ were cooled at 0 °C in an ice-water bath and immediately centrifuged at 6000xg and 0 °C for 10 min. Pellets were suspended in 50% v/v methanol (0.01 g_cdw_/mL) and incubated at 70 °C for 30 min. The supernatant was ultrafiltered on 3 kDa centricon devices and finally dried in a vacuum centrifuge before HPCE analysis.

### Small scale hydrolysis tests

Ultrafiltered fermentation supernatants at concentrations ranging from 6 to 20 g/L of K4 CPS were hydrolysed at pH 2.8, by adding hydrochloric acid 12 M, and 90 °C. Reactions were incubated for 3, 5 and 10 min to evaluate defructosylation over time. After incubation the reaction was stopped immediately by adjusting the pH to 7 with NaOH and lowering the temperature on ice.

Hydrolysis tests on low molecular weight (235 ± 13 kDa) hyaluronic acid (LHA) were carried out in the same conditions to investigate Mw variations. Samples with a concentration of 14 ± 2 g/L of LHA, were incubated for 5, 10, 15 and 30 min to study the decrease of Mw over time.

After hydrolysis HA samples were analysed by SEC-TDA for the Mw determinations, whereas K4 CPS samples were analysed by capillary electrophoresis. Tests were carried out at least three times.

### Purification strategy

After all fermentation experiments the biomass (waste) was collected by centrifugation at 4 °C and 5000 × g for 30 min, and the supernatant containing the capsular polysaccharide was collected and microfiltered through 0.65 μm Sartopure PP2 midicap (Sartorius Stedim, Gottingen, Germany) and treated with 10 U/L of proteases from *Aspergillus oryzae* (Sigma-Aldrich, Missouri, USA). The supernatant was tenfold concentrated and diafiltered by tangential flow filtration on a Uniflux-10, (GE Healthcare, Illinois, USA). Polyethersulfone membranes (GE Healthcare, Illinois, USA), with a nominal molecular mass cut-off of 30 kDa and a total membrane surface area of 0.1 m^2^ were used in all filtration steps. The parameters set for the process were a flux of 4.5 L/h and a transmemebrane pressure (TMP) of 0.6 bar. The obtained retentate was hydrolysed at 90°, pH 2.8 ± 0.1, reached through the addition of HCl 12 M, for 10 min. The reaction was stopped by adding NaOH 20 M to increase the pH to 7, and by reducing the temperature to 25 °C.

**Figure 1:**
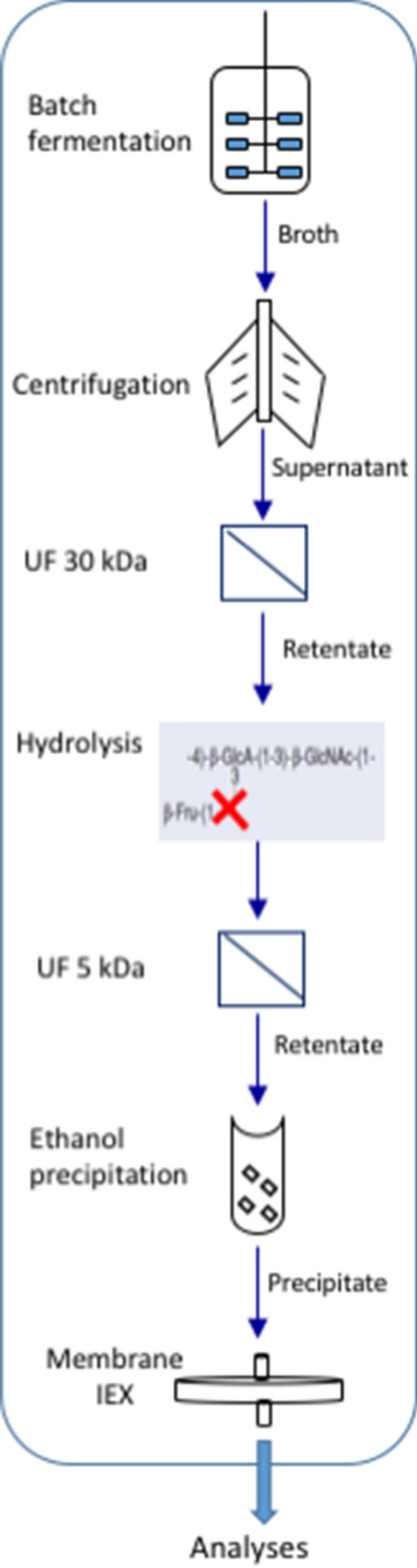
Strategy applied for the defructosylation and purification of the CPS from the broths obtained after fed-batch fermentations from all strains.

A second UF step was next conducted on 5 kDa membranes. The retentate was precipitated with 3 V of cold ethanol 96% (v/v) and a conductivity of about 13–15 mS/cm by letting aggregates sediment o/n. The obtained precipitate was dried in a vacuum oven at 40 °C over-night and redissolved in de-ionized water. The defructosylated-polysaccharides from all strains were further purified by weak anion exchange chromatography on small diethylamine (DEAE) resin devices (Vivapure D maxi H, Sartorius Stedim), as previously described^18^. The fractions eluted with 250–300 and 350 mM NaCl were dialyzed and characterized by SEC-TDA and capillary electrophoresis. The purification strategy used is depicted in Fig. 1.

### SEC-TDA molecular weight determination

Molecular weight analyses of the purified chondroitin and CPS K4 capsular polysaccharide fractions, obtained from the anion exchange chromatography, were performed by a high performance size exclusion chromatographic system (Malvern, UK), equipped with a triple detector array module including a refractive index detector (RI), a four-bridge viscosimeter (VIS), and a laser detector (LS) made of a right-angle light scattering (RALS) detector and a low-angle light scattering (LALS) one, as previously reported^21^. Two gel-permeation columns (TSK-GEL GMPWXL, 7.8 × 30.0 cm, Tosoh Bioscience, Italy) equipped with a guard column, were set in series to perform the analyses. The OmniSEC software program was used for the acquisition and analysis of the data. Elution was performed in isocratic conditions with 0.1 M NaNO_3_ at pH 7.0, at a flow rate of 0.6 mL/min, at 40 °C in 50 min runs, after injection of 0.1 mL of the sample. The calibration of the instrument was performed by using a polyethylene oxide (PEO) standard (22 kDa PolyCAL, Viscotek, Malvern). Samples in a concentration range between 0.4 and 2.5 g/L were analyzed in duplicate. Specific evaluation of the CPSs and chondroitin average molecular weight, of the polidispersity index (Mw/Mn) and of the intrinsic viscosity (IV) were determined by all the detector signals applying the equations reported by the manufacturer (data from Viscotek) and on the basis of the CPS dn/dc values.

### Transmission electron microscopy

Broth samples of *E.coli* K4 wild type, and recombinant strains, from the exponential growth phase were fixed in 2.5% (w/v) glutaraldehyde, postfixed in 1% OsO_4_ and Uranil Acetate Replacement (Electron Microscopy Science), de-hydrated in ethanol and embedded in epoxy resin. Ultrathin sections (60 nm) were obtained, contrasted in aqueous lead-hydroxide solution followed by Tannic Acid treatment, and photographed by Zeiss-LIBRA120 TEM. Measurements of the width of the periplasmic space were obtained using ITem Zeiss software. More than 30 measurements were performed for each strain and data significance was determined by two tailed non homoscedastic t-student analyses.

## Results

### Fed-batch fermentations

The wild type *E.coli* K4 and all recombinant strains were grown in 2 L and 22 L bioreactors in fed-batch conditions to analyze CPS production. After about 6–7 h of growth all the glucose was exhausted, as indicated by the pO_2_ increase, and a concentrated feed was added to prolong growth for up to 23 h to increase biomass and polysaccharide production. Time course of fed-batch experiments is reported in the Supplementary Figures S2 and S3.

Data generated in the two experimental set-ups were averaged separately and results are reported in Table 2. Statistical significance was determined by applying a two tailed non homoscedastic t-test between the wild type and each recombinant strain (Table 2). As indicated by capillary electrophoresis analyses, all recombinant strains demonstrated an increased production of CPS ranging on average from about 1.5 to 2.3 fold (Table 2), compared to the wild type; moreover, genetic modifications also led to improved yields (Y_p/s_, Y_p/x_) and process productivities (r_p_) on both fermentation scales while the yield of biomass on substrate (Y_x/s_) was not affected in the recombinant strains. No significant differences were found among experiments conducted on the 2 and 22 L reactors. Only the wild type strain showed a slight improvement of the polysaccharide titers on the 22 L fermenter, however, by applying a two tailed non-homoscedastic t-test a statistical significance was not found.

**Table 2:** Results obtained from fed-batch fermentations conducted on the 2 and 22L bioreactors.

Bioreactor	Strain	CPS (g/L)	ODmax	Y_x/s_ (ODmax/g)	Y_p/s_ (mg/g)	Y_p/x_ (mg/OD_max_)	r_p_ (mg/Lh)
2L	*E.coli* K4 wt	0.84 ± 0.10	34 ± 3	0.67 ± 0.02	16 ± 2	24 ± 1	37 ± 4
	EcK4rv_1	1.39 ± 0.04*	30 ± 4	0.56 ± 0.16	25 ± 1***	45 ± 4*	60 ± 2**
	EcK4rv_2	1.91 ± 0.10***	31 ± 2	0.62 ± 0.04	39 ± 2**	61 ± 1***	83 ± 4***
	EcK4rv_3	1.84 ± 0.12***	34 ± 3	0.60 ± 0.04	35 ± 3**	56 ± 7*	80 ± 5**
	EcK4rv_5	2.14 ± 0.10***	31 ± 2	0.58 ± 0.04	41 ± 6**	70 ± 12*	93 ± 4***
22L	*E.coli* K4 wt	1.06 ± 0.10	30 ± 2	0.63 ± 0.05	21 ± 3	34 ± 5	45 ± 5
	EcK4rv_1	1.34 ± 0.09*	30 ± 4	0.61 ± 0.07	28 ± 2**	44 ± 4*	58 ± 4*
	EcK4rv_2	2.00 ± 0.29*	30 ± 1	0.55 ± 0.02	36 ± 5**	66 ± 8*	87 ± 12*
	EcK4rv_3	1.69 ± 0.08**	32 ± 1	0.62 ± 0.05	35 ± 2**	52 ± 4**	74 ± 6**
	EcK4rv_5	2.09 ± 0.19**	30 ± 4	0.61 ± 0.04	44 ± 5**	72 ± 12*	91 ± 8**

Data are mean values and standard deviations of at least 3 experiments performed on each scale. Statistical analyses:*p < 0.05; **p < 0.01; ***p < 0.0005, indicate difference significance in respect to the wild type sample.

CPS, capsular polysaccharide; Y_x/s_, biomass (OD_max_) obtained per g of substrate (glucose) consumed; Y_p/s_, mg of CPS produced per g of substrate consumed; Y_p/x_, mg of CPS produced per biomass (OD_max_); r_p_, volumetric productivity calculated as the mg of CPS produced per L per h; CPS concentration after 23 h (last point) was considered for the calculation.

**Figure 2:**
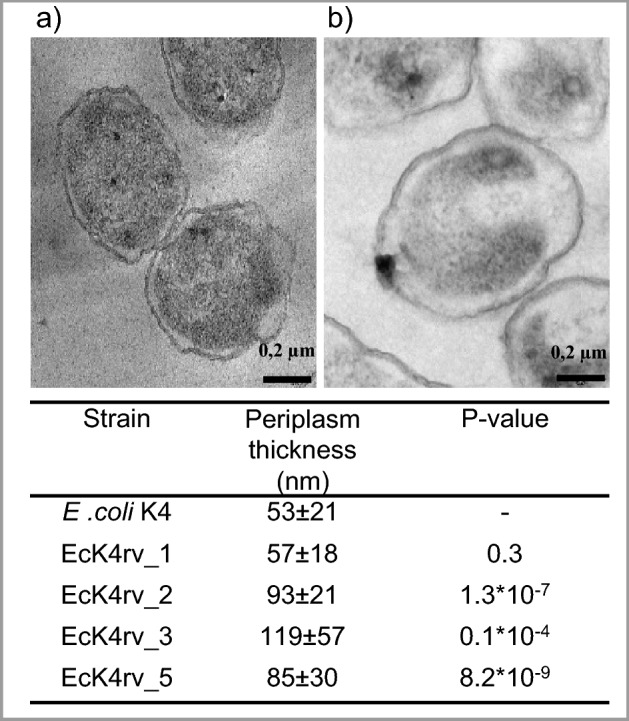
TEM images of (**a**) *E. coli* K4 WT, (**b**) EcK4rv_5 strains. The values reported in the table correspond to the width of the periplasmic space in the different strains. Mean and standard deviation were obtained by over 30 measurments for each strain. Data significance was determined by applying a two tailed non homoscedastic t-test between the wild type and each recombinant strain.

In order to highlight potential morphologic changes among the wt and the mutant strains, the latter were also investigated at the microscopic level, and unexpectedly differences in periplasmic space size compared to the wild type were identified, as observed in Fig. 2. In particular, EcK4rv_2, EcK4rv_3 and EcK4rv_5 demonstrate a 1.8, 2.2 and 1.6 fold wider region compared to the wild type, respectively, whereas no difference was found for strain EcK4rv_1.

### Defructosylation and Mw reduction during hydrolysis

**Figure 3:**
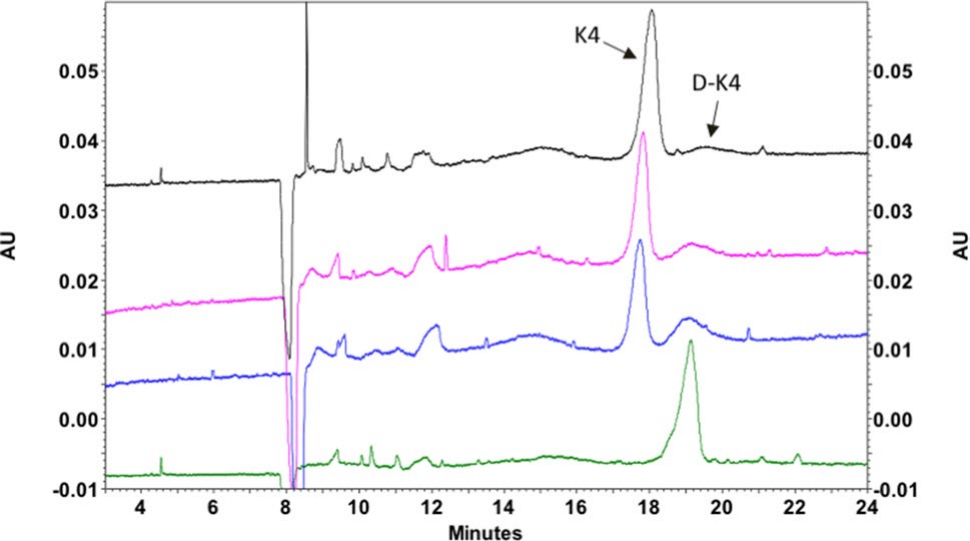
Capillary electrophoresis analyses of K4 and defructosylated K4 (D-K4) before and after hydrolysis. The reaction was performed at pH 2.8 and 90 °C. The figure shows the progressive defructosylation of a sample of polysaccharide containing the highest ratio of K4 to D-K4. Black line: pre-hydrolysis sample; Pink line: 3′ post-hydrolysis; blue line: 5′ post-hydrolysis; green line: 10′ post-hydrolysis.

**Figure 4:**
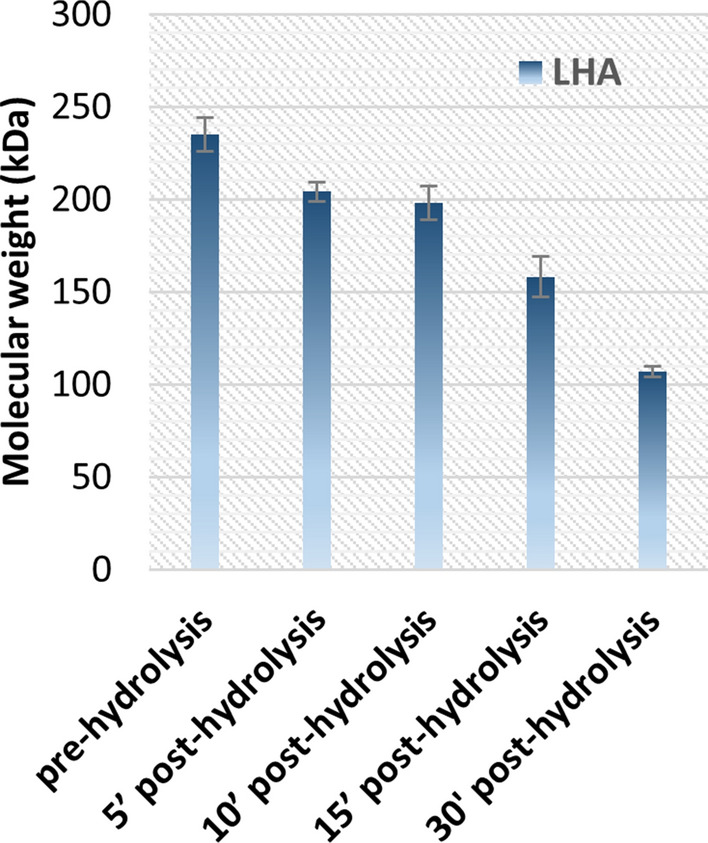
Hydrolysis of LHA at 90 °C and pH 2.8. Mw was determined by SEC-TDA analyses.

A hydrolytic step is necessary to remove the fructose residue in order to convert K4 CPS into (unsulphated) chondroitin. The incubation time required to defructosylate K4 CPS samples produced by all the recombinant strains was evaluted by incubating the supernatants at 90 °C and pH 2.8 for 3, 5, and 10 min. The percentage of polymer fructosylation before hydrolysis ranged from 40 to 90%. The assay was optimized on a sample containing the highest percentage of fructosylated polymer (90%). Results indicated that after 3 and 5 min of incubation only 14 and 22% of the samples were defructosylated, whereas after 10′ of incubation, the fructose was completely eliminated (Fig. 3). Hydrolysis was also performed on pure low molecular weight HA (LHA) to evaluate molecular weight reduction over time, and results are reported in Fig. 4.

As expected, small scale trials demonstrated an increased depolymerization with growing incubation times, that led to an almost 60% reduction of the initial Mw after 30′ in the tested conditions. After 10 min of incubation the chain length decreased by 19 ± 3%. Higher incubation times (15 and 30 min) also resulted in a significant increase of the polydispersity index, that reached a value of about 2.1 ± 0.1 in these samples.

### Chondroitin purification and Mw determination

The purification procedure applied in this work, necessary to remove the fructose residue and part of the LPS to allow accurate Mw determination, was initially performed on about 1.8 L of supernatant recovered from the small scale reactors; then it was scaled up to process approximately 12 L of supernatant for each strain. The purification steps allowed to obtain a sample purity ranging between 93 and 96%. During the course of the process, concentrated samples were analyzed by capillary electrophoresis to follow the decrease of the LPS contamination, and also by SEC-TDA (that has a higher sensitivity). For all strains, initial UF steps resulted quite similar in terms of TMP, that slightly increased from 0.6 to 0.8 bar, and flux that decreased from about 4.5 to 3.8 L/h. After the UF and hydrolytic step the LPS contamination was still above the lower detection limit (data not shown) therefore further purification steps, based on an ethanol precipitation and following anion exchange chromatography, were necessary to lower the LPS concentration and obtain a reliable evaluation of the Mw of the polymers produced by all strains. As shown in Table 3, after purification, the molecular weight of the polysaccharide produced by EcK4rv_2, EcK4rv_3 and EcK4rv_5 was 2.3, 2.6, and 1.8 fold higher, respectively, compared to that produced by the wt strain. In EcK4rv_1, the polysaccharide size was not increased.

**Table 3:** Molecular weight determined by SEC-TDA analyses of the polysaccharide produced by the wild type *E. coli* K4 and the recombinant strains investigated in this study. Mw/Mn indicates the polydispersity index.

Strain	Mw (kDa)	Mw/Mn
*E.coli* K4 wt	52 ± 5	1.10 ± 0.07
EcK4rv_1	47 ± 5	1.18 ± 0.06
EcK4rv_2	130 ± 17*	1.35 ± 0.15
EcK4rv_3	133 ± 18*	1.24 ± 0.04
EcK4rv_5	101 ± 20*	1.17 ± 0.02

Samples were obtained by purifying the fermentation supernatants with the protocol described in the “Materials and methods” section.

*Indicates p < 0.05 when comparing the wild type with the recombinant strains by means of a two tailed non homoscedastic t-student analysis.

### Determination of UDP-sugar levels

**Figure 5:**
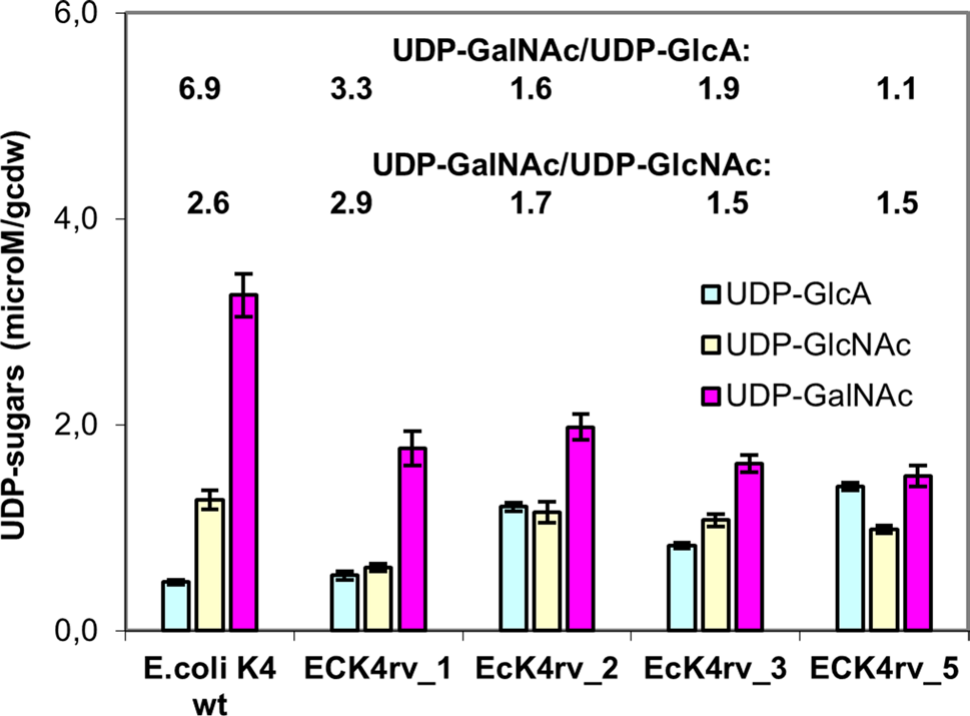
UDP-sugar pools in *E. coli* K4 and in the recombinant strains. Biomass samples were collected during fed-batch experiments in the mid exponential phase (3 h of growth); UDP-sugars were extracted with hot methanol and analyzed by capillary electrophoresis.

Biomass samples of wild type and recombinant strains withdrawn during the mid-exponential growth phase were boiled in hot methanol and the extracted UDP-nucleotide sugars were quantified by capillary electrophoresis (Fig. 5). As shown in the figure all pools were affected by the genetic modifications although to different extents. In particular, compared to the wild type UDP-GalNAc decreased in all recombinant strains by on average 47 ± 6% without a significant correlation with any particular metabolic engineering strategy. Interestingly, with the exception of EcK4rv_1, a 38 ± 4% lower UDP-GalNAc/UDP-GlcNAc ratio was observed in all the other recombinant strains compared to the wild type; moreover, compared to the latter all strains demonstrated also a lower UDP-GalNAc/UDP-GlcA ratio with a decrease of about 52 to 84%. Higher concentrations of UDP-GlcA were found in EcK4rv_2, EcK4rv_3 and EcK4rv_5, respectively, with an increase from 1.7 to 3-fold, whereas this metabolite was only slightly changing in EcK4rv_1 (13%).

## Discussion

Novel potential fields of application of chondroitin and CS are emerging, and mainly due to limitations in manufacturing of higher molecular weight polymers, the prospective of using such material has never been investigated so far. One strategy to obtain tailor made longer polysaccharide chains is by utilizing recombinant microorganisms, and to develop a purification protocol that does not impact the polymer weight with harsh operating conditions and that also allows to analyze the molecular weight in a highly accurate way.

In order to be able to investigate potential differences in molecular weight of the polymers, produced by *E.coli* K4, we developed a preliminary purification process that allowed (1) polymer defructosylation without strongly affecting its molecular weight, and (2) the reduction of the LPS content to allow reliable molecular weight determination through SEC-TDA.

By using HA as model system, we initially demonstrated that an already attenuated protocol of a previously developed hydrolysis step for the defructosylation and LPS removal^22^, significantly reduced the molecular weight (up to 60% already at 30 min), and also that this can be mitigated by reducing the time and temperature of incubation.

Subsequent ultra-filtration and ethanol precipitation were followed by an IEX chromatography step. This step was required to reduce the LPS content that otherwise may interfere with the SEC-TDA analyses leading to misguiding results. SEC-TDA allows to establish more accurately the chondroitin Mw also in the absence of a chondroitin standard compared to previously reported methods, such as SEC-LLS^23^.

This protocol was then used for the analysis of *E.coli* K4, a natural producer of CS-like polysaccharide, and four recombinant derivates thereof, overexpressing endogenous genes involved in the biosynthesis of UDP-sugar CPS precursors (Table 1). The construction and a very basic characterization of all these strains with respect to CPS production levels were already previously described^12^. In this study we extended the physiological characterization of these strains in well-controlled bioreactors also to larger scales (up to 22 L), as it has been already reported that different cultivation parameters like fermentation duration, but also in particular scale dependent factors like mixing can impact the molecular weight of the produced biopolymers^13,14^. From the results (Table 2) it can be seen that the enhanced biopolymer productivity of basically all recombinant strains is confirming previously reported results^12^, and also that there does not seem to be a significant impact on this overproduction up to a bioreactor scale of 22 L.

More interestingly, by using the above described purification protocol and analytical method, three of the four recombinant strains (those overexpressing *kfoF*) showed a highly significant increase (more than 50%, see Table 3) of the molecular weight of the overproduced CPS, once again clearly demonstrating the importance of precursor supply not only for its production, but also its potential for controlling and tailoring the molecular weight of the biopolymer. Overall UDP-glucose dehydrogenase seems to have a key role not only in enhancing CPS titers but also in increasing their chain length.

Several studies evaluated the effects of metabolic engineering strategies on UDP-sugar changes in *E. coli* K4^12,20,24,25^, whereas in *Streptococcus* the effect of changes in UDP-sugar pools was also investigated in relation to HA Mw^26–29^. Chen et al.^30^ found a strong correlation between higher UDP-GlcNAc levels and higher Mw, this association is however lost when a concentration threshold is reached, indicating the influence of other factors. Therefore, also the UDP-sugar pools in *E. coli* K4 as well as it’s recombinant derivates were investigated here, in order to identify potential correlations to CPS production and its molecular weight. UDP-sugars extracted from exponentially growing cells in the batch phase showed that, compared to the wild type and to EcK4rv_1 (overexpressing only *kfoA*), the other recombinant strains showed lower UDP-GalNAc/UDP-GlcA ratios (ranging between 1 and 2) and also lower ratios of UDP-GalNAc/UDP-GlcNAc. These are mainly due to the increased production of UDP-GlcA, compared to the wild type, and to lower concentrations of UDP-GalNAc (that are very similar in all recombinant strains) that is maybe used to support higher CPS titers.

Data presented here, seem to indicate that reducing the concentration delta among UDP-GalNAc and UDP-GlcA during the exponential phase allows to increase the size of K4CPS produced, and that one of the potential strategies to do so is by overexpressing *kfoF.*

Finally, potential physical changes in cell morphology due to the overproduction of CPS in the recombinant strains were addressed. During experiments biomass samples collected in the late exponential growth phase, where fixed and analysed by TEM. Interestingly, the staining method highlighted a periplasmic space with increased thickness in all strains overexpressing *kfoF*.

The periplasm has several roles in bacterial cells^31^ among which also the secretion of the LPS and of the CPS. To the best of our knowledge the only observations of enlarged periplasmatic space have been linked to mutations increasing the length of Lpp (Braun’s lipoprotein), which covalently links the peptidoglycan to the outer membrane^31,32^. Interestingly these mutations have also been studied in relation to the production of capsular polysaccharide through the RCS-signaling pathway, that senses external damage/disorder and remodels the bacterial surface. However, we considered unlikely that the RCS pathway is involved in the increased CPS production that we observe, since this would require also a second mutation that enlarges RCS-F, another regulator of capsule biosynthesis^33^. Therefore, a crosstalk between capsule molecular weight and periplasmic space width was highlighted in this study, however further studies are necessary to increase knowledge on the biological meaning of this relationship.

## Conclusions

Overall this study exploited efficient fed-batch processes for demonstrating how the genetic modifications in the precursor UDP-sugar biosynthesis pathways lead not only to higher titers of capsular polysaccharide but also to polymers with increased molecular weight.

The chemical modification of biopolymers often requires harsh conditions that might reduce their molecular weight e.g. the conversion of chondroitin into CS^34,35^. Thereof, the production of chondroitin with higher Mw is interesting not only to investigate potential novel properties but also to facilitate the obtainment of its semi-synthetic derivatives.

## Supplementary information

Supplementary Information.
