# Supplementary material for: Production and purification of higher molecular weight chondroitin by metabolically engineered Escherichia coli K4 strains
Source: Sci Rep. 2020 Aug 6;10:13200. doi: 10.1038/s41598-020-70027-9 (PMC7411012; doi:10.1038/s41598-020-70027-9)
Supplement: Supplementary file 1 — Supplementary Information. [file 41598_2020_70027_MOESM1_ESM.docx]

**Production and purification of higher molecular weight chondroitin by metabolically engineered *Escherichia coli* K4 strains**

S. D’ambrosio, A. Alfano, E. Cassese, O.F. Restaino, S. Barbuto Ferraiuolo, R. Finamore, M. Cammarota, C. Schiraldi^§^, D. Cimini^§^*.

*Department of Experimental Medicine, Section of Biotechnology and Molecular Biology, University of Campania L.Vanvitelli, via de Crecchio 7, 80138 Napoli, Italy.*

^§^These authors equally contributed to the work

*Corresponding author: donatella.cimini@unicampania.it

Supplementary Figure S1- Feeding profile used for all fed-batch fermentation experiments.

Supplementary Figure S2-Time course of fed-batch fermentations on 2L bioreactors. The curves indicate biomass production (OD_600_ nm) and glucose consumption over time for the wild type *E.coli* K4 and the recombinant strains. Data are mean and standard deviations of three separate experiments.

Supplementary Figure S3-Time course of fed-batch fermentations on 22L bioreactors. The curves indicate biomass production (OD_600_ nm) and glucose consumption over time for the wild type *E.coli* K4 and the recombinant strains. Data are mean and standard deviations of three separate experiments.
